# Phylogenomic investigation of an outbreak of fluoroquinolone-resistant *Salmonella enterica* subsp. *enterica* serovar Paratyphi A in Phnom Penh, Cambodia

**DOI:** 10.1099/mgen.0.000972

**Published:** 2023-03-24

**Authors:** Rutaiwan Dusadeepong, Gauthier Delvallez, Sokleaph Cheng, Soda Meng, Navin Sreng, Joanne Letchford, Kimcheng Choun, Syna Teav, Liselotte Hardy, Jan Jacobs, Tuyet Hoang, Torsten Seemann, Benjamin P. Howden, Philippe Glaser, Timothy P. Stinear, Koen Vandelannoote

**Affiliations:** ^1^​ Bacterial Phylogenomics Group, Pasteur Institute of Cambodia, Phnom Penh, Cambodia; ^2^​ Medical Biology Laboratory, Pasteur Institute of Cambodia, Phnom Penh, Cambodia; ^3^​ LMI Drug Resistance in South East Asia, Pasteur Institute of Cambodia, Phnom Penh, Cambodia; ^4^​ Laboratory of Environment and Food Safety, Pasteur Institute of Cambodia, Phnom Penh, Cambodia; ^5^​ Diagnostic Microbiology Development Program, Phnom Penh, Cambodia; ^6^​ Infectious Disease Department, Sihanouk Hospital Center of HOPE, Phnom Penh, Cambodia; ^7^​ Laboratory Unit, Sihanouk Hospital Center of HOPE, Phnom Penh, Cambodia; ^8^​ Department of Clinical Sciences, Institute of Tropical Medicine, Antwerp, Belgium; ^9^​ Department of Microbiology, Immunology and Transplantation, KU Leuven, Leuven, Belgium; ^10^​ Microbiological Diagnostic Unit Public Health Laboratory, Department of Microbiology and Immunology, Doherty Institute for Infection and Immunity, University of Melbourne, Melbourne, Victoria, Australia; ^11^​ Centre for Pathogen Genomics, Department of Microbiology and Immunology, Doherty Institute for Infection and Immunity, University of Melbourne, Melbourne, Victoria, Australia; ^12^​ Evolution and Ecology of Antibiotic Resistance Unit, APHP–CNRS–UMR6047, Institut Pasteur–University Paris-Saclay–Université Paris Cité, Paris, France

**Keywords:** antimicrobial resistance, Cambodia, phylogenomics, *Salmonella enterica *Paratyphi A, whole-genome sequencing

## Abstract

In early 2020, the Medical Biology Laboratory of the Pasteur Institute of Cambodia isolated an unusually high number of fluoroquinolone-resistant *Salmonella enterica* subspecies *enterica* serovar Paratyphi A strains during its routine bacteriological surveillance activities in Phnom Penh, Cambodia. A public-health investigation was supported by genome sequencing of these Paratyphi A strains to gain insights into the genetic diversity and population structure of a potential outbreak of fluoroquinolone-resistant paratyphoid fever. Comparative genomic and phylodynamic analyses revealed the 2020 strains were descended from a previously described 2013–2015 outbreak of Paratyphi A infections. Our analysis showed sub-lineage 2.3.1 had remained largely susceptible to fluoroquinolone drugs until 2015, but acquired chromosomal resistance to these drugs during six separate events between late 2012 and 2015. The emergence of fluoroquinolone resistance was rapidly followed by the replacement of the original susceptible Paratyphi A population, which led to a dramatic increase of fluoroquinolone-resistant blood-culture-confirmed cases in subsequent years (2016–2020). The rapid acquisition of resistance-conferring mutations in the Paratyphi A population over a 3 year period is suggestive of a strong selective pressure on that population, likely linked with fluoroquinolone use. In turn, emergence of fluoroquinolone resistance has led to increased use of extended-spectrum cephalosporins like ceftriaxone that are becoming the drug of choice for empirical treatment of paratyphoid fever in Cambodia.

## Data Summary

Newly sequenced short-read data are available from the European Nucleotide Archive (ENA), associated with study accession number PRJEB57163 (https://www.ebi.ac.uk/ena/browser/view/PRJEB57163). Run accession numbers for individual samples are given in Table S1 (available with the online version of this article).

**Impact Statement**

Paratyphoid fever, caused by *Salmonella enterica* subspecies *enterica* serovar Paratyphi A, is a severe systemic febrile illness restricted to the human host that is transmitted via the faecal–oral route. Fluoroquinolones are the first choice for oral treatment of non-complicated enteric fever. Here, we used comparative genomics to investigate an unusual increase in the number of fluoroquinolone-resistant Paratyphi A strains isolated from patients in Phnom Penh, Cambodia, in 2020. We sequenced and compared the genomes of these Paratyphi A outbreak strains with an international isolate panel that represents the breadth of the serovar’s lineage 2.3 diversity. Our comparative genomic analysis revealed the 2020 outbreak strains were descended from a previously described 2013–2015 Cambodian outbreak of lineage 2.3.1 Paratyphi A infections. Our analysis also showed Cambodian sub-lineage 2.3.1 remained largely susceptible to fluoroquinolone drugs until 2015, and acquired chromosomal resistance to these drugs during six separate events between late 2012 and 2015. These results are suggestive of a strong selective pressure on the Cambodian Paratyphi A population linked with fluoroquinolone use. Our findings point towards the consequences of uncontrolled and widespread use of fluoroquinolones, which until now have been the most effective drugs available for the management of enteric fever.

## Introduction

*Salmonella enterica* subspecies *enterica* serovars Typhi and Paratyphi A (referred to as Typhi and Paratyphi A hereafter) are closely related Gram-negative bacteria that are restricted to the human host. Both serovars cause clinically similar syndromes of prolonged, severe, systemic salmonellosis that are referred to as typhoid fever and paratyphoid fever, although both are also known under the generic designation ‘enteric fever’ [1]. At present, enteric fever is estimated to be responsible for an annual global burden of approximately 14.3 million cases and 136 000 deaths [2]. Transmission of both serovars occurs through faecal and urine contamination of food or water, with an incubation period of up to 1 month. During the late 20th century, disease incidence in high-income countries dropped dramatically as sanitation infrastructure and hygiene practices improved [3], while incidence remained high in low-resource settings. At present, the highest global incidence rates are observed in Southern Asia [approx. 549.2 (480.7 to 625.4)/100 000 person-years] [2].

While historically, enteric fever was mostly caused by Typhi, the relative importance of Paratyphi A has been rising in the last 23 years, in particular in Southern Asia [4–6]. Antibiotics currently represent the central pillar of Paratyphi A disease control in endemic regions, as there are no effective vaccines available against the serovar [7]. The antibiotics ampicillin (AMP), chloramphenicol (CHL) and trimethoprim–sulfamethoxazole (TRS) were used as first-line agents against enteric fever until the 1980s, when the indiscriminate use of these conventional anti-typhoid drugs led to the appearance of plasmid-mediated antibiotic resistance and the first AMP, CHL, TRS multidrug resistant (MDR) cases [8]. By the 1990s, fluoroquinolones like ciprofloxacin (CIP) were widely adopted and remain the recommended first-line treatment for uncomplicated enteric fever, including MDR cases [9]. Relative to Typhi, serovar Paratyphi A isolates have predominantly remained susceptible to antibiotics until the turn of the 21st century [5, 10], when Southern Asia started reporting both MDR Paratyphi A cases [11, 12] and isolates with reduced susceptibility to fluoroquinolones [13–15].

Between January and September 2020, the Medical Biology Laboratory of the Pasteur Institute of Cambodia (Institut Pasteur du Cambodge) (MBL-IPC) isolated an unusually high number of fluoroquinolone-resistant Paratyphi A strains during its routine diagnostic microbiology activities in Phnom Penh, the capital of Cambodia. The MBL-IPC reported these findings to the Cambodian Centers for Disease Control and Prevention (CDC), which requires notification from laboratories when three or more laboratory confirmed cases of paratyphoid fever are discovered within a 1 week period. When various other clinics and hospitals dotted in and around Phnom Penh started notifying increasingly more cases to the Cambodian CDC in 2020, the MBL-IPC supported a public-health investigation by sequencing and comparing the genomes of all MBL-IPC Paratyphi A strains to gain insights into the genetic diversity and population structure of this early 2020 outbreak of Cambodian fluoroquinolone-resistant paratyphoid fever. We compared the genomes of the studied outbreak to all publicly available whole-genome sequenced Paratyphi A strains to interpret the evolution and the possible origin of the outbreak. Moreover, we attempted to trace potential point sources of the paratyphoid outbreak by screening a collection of *Salmonella* spp. strains isolated from Phnom Penh food samples by the Laboratory of Environment and Food Safety of the Pasteur Institute of Cambodia (Institut Pasteur du Cambodge) (LEFS-IPC).

## Methods

### Bacterial isolates

A panel of 32 *S. enterica* serovar Paratyphi A strains were isolated between January and September of 2020 at the MBL-IPC from patients who presented at various public and private hospitals/clinics in Phnom Penh, Cambodia (Table S1). This panel represents 100 % of Paratyphi A strains identified by the MBL-IPC during this period. The MBL-IPC isolates were grown from blood cultures using the BacT/Alert system (bioMérieux) and subsequently subcultured on Oxoid chocolate agar (ThermoFisher Scientific). All bacterial isolates had previously been assigned to the serovar Paratyphi A using MALDI-TOF MS (Bruker Daltonics) and serotyping with commercial antisera (Bio-Rad) according to the White–Kauffmann–Le Minor classification scheme [16]. All isolates were maintained for prolonged storage at ≤−80 °C in Difco skim milk (Becton Dickinson) medium and glycerol.

Additionally, we screened a collection of 83 *Salmonella* spp. strains that the LEFS-IPC isolated from various food samples originating from Phnom Penh and the surrounding provinces between 2018 and 2020. The LEFS-IPC performs routine microbiological control of food for various commercial clients that are mainly located in Phnom Penh. As these *Salmonella* spp. strains had previously only been identified to the genus level, we performed a set of Typhi- and Paratyphi-specific gene combination quantitative PCR (qPCR) assays directly on crude DNA extracts of bacterial colonies to identify serovar Paratyphi A strains within this collection [17].

### Antibiotic-susceptibility testing (AST)

Phenotypic AST of Paratyphi A isolates was performed using both broth microdilution and disc diffusion, in accordance with the European Committee on Antimicrobial Susceptibility Testing (EUCAST) 2021 guidelines [18]. *Escherichia coli* ATCC 25922 (serotype O6), a recommended reference strain for AST of *Enterobacterales *, was used as a control. Minimum inhibitory concentrations (MICs) were determined using custom-designed Sensititre AST plates (ThermoFisher Scientific) assaying for the following antibiotics: AMP, cefixime (CIX), ceftazidime (CTZ), cefotaxime (CTA), ceftriaxone (CTR), cefepime (CEP), meropenem (MEM), gentamicin (GEN), azithromycin (AZI), CIP, gatifloxacin (GAT), TRS and CHL. Disc diffusion was performed on Mueller–Hinton agar with 5 µg pefloxacin (PEF) discs (Rosco Diagnostica).

### Whole-genome sequencing (WGS)

The 32 Paratyphi A strains isolated by the MBL-IPC were selected for Illumina sequencing. Genomic DNA (gDNA) was obtained by harvesting two loopfuls of colony-purified, plated isolates, followed by heat inactivation, enzymatic digestion and DNA purification using the GenElute bacterial genomic DNA kit (Sigma-Aldrich). Paired-end index-tagged sequencing libraries were prepared from gDNA extracts with the Nextera XT DNA library preparation kit. Genome sequencing was performed on an Illumina NextSeq 500 sequencer according to the manufacturer's protocols, with 150 bp paired-end sequencing chemistry. We used the preprocessing tool Trimmomatic v0.39 [19] to: trim residual sequencing adaptors, crop the 3' end of reads where quality monotonically decreases, and filter Illumina reads.

### Sequence read data acquisition

In addition to the 32 clinical isolates, a selection of published Paratyphi A lineage 2.3 (formerly known as lineage C) paired-end Illumina sequence reads was included in our analysis to provide appropriate genetic context for interpreting the evolution and diversity of the studied outbreak of enteric fever. We used Treemmer v0.3 [20] to evaluate the redundancy of a maximum-likelihood (ML) phylogenetic tree (see below) of all publicly available Paratyphi A lineage 2.3 sequence reads and reduce its complexity by eliminating leaves that contribute the least to the tree’s overall diversity. Leaves representing Cambodian isolates were excluded from this pruning step. This allowed us to down sample all publicly available read-sets to a manageable subset that still reflects the breadth of the serovar’s lineage 2.3 diversity. All read-sets were obtained from the National Center for Biotechnology Information (NCBI) Sequence Read Archive (SRA) and are detailed in Table S1.

### Paratyphi A genotyping

We used the Paratype v1_beta2 [21] SNP-based genotyping scheme in fastq mode to assign isolates to genotypes.

### *De novo* assembly and *in silico* detection of antimicrobial resistance (AMR)

Illumina reads were *de novo* assembled into contigs using Shovill v1.1.0 [22], a pipeline that uses the SPAdes genome assembler v3.15.3 at its core. AMRFinderPlus v3.10.14 [23] and its accompanying database v2021-12-21.1 were used with default parameters to find acquired AMR genes and point mutations in assembled nucleotide sequences.

### Read alignment and SNP detection

Read mapping and SNP detection were performed using the Snippy v4.6.0 pipeline [24]. Here, the Burrows–Wheeler Aligner (BWA) v0.7.17 [25] was used to map read-pairs to the Paratyphi A strain ATCC 9150 reference genome (GenBank accession no. CP000026). Mean read depths and mean read lengths of mapped reads were determined with SAMtools v1.4 [26], and are summarized for all isolates in Table S1. SNPs were subsequently identified using the variant caller FreeBayes v1.3.5 [27] with: the minimum read mapping quality to consider in variant calling as 60, the minimum quality a nucleotide needs to be used in variant calling as 13, the minimum number of reads covering a position to be considered as 10, and the minimum proportion of reads that must differ from the reference at a position as 0.9. Snippy was used to pool all identified SNP positions called in at least one isolate and interrogate all isolates of the panel at that position to generate a multiple sequence alignment of ‘core SNPs’.

### Recombination/prophage detection

Artificial whole-genome sequences were constructed for each isolate by changing SNP positions in the ATCC 9150 reference genome with the consensus allele identified by the Snippy pipeline at that position in that isolate. These sequences were combined into an artificial whole-genome sequence alignment that was fed into the Gubbins v3.0.0 [28] recombination detection pipeline. We ran Gubbins with default parameters to identify all recombinant regions in the bacterial chromosome. Similarly, we used phaster [29] to detect all horizontally acquired functional and non-functional bacteriophage genes in the ATCC 9150 reference genome. Our multiple sequence alignment of core SNPs was filtered down by excluding all variation detected in recombinant and prophage regions to obtain an alignment of purely vertically acquired mutations.

### Haplotype network inference

We used PopART v1.7 [30] to visualize the relationships between individual haplotypes at the population level in networks. Networks were inferred using the median-joining network method [31].

### ML phylogenetic analysis

A ML phylogenetic tree was inferred from the filtered core-SNP alignment using iq-tree v.2.1.2 [32] with the MFP+ASC model, which includes SNP ascertainment bias correction and automatically selects the best-fit substitution model using ModelFinder [33]. Branch support for the ML phylogeny was assessed with 1 000 standard nonparametric bootstrap analyses.

### Bayesian phylogenomic analysis

We used beast2 v2.6.7 [34] to infer a time-tree and estimate divergence times of *Salmonella* Paratyphi A lineage 2.3. The BEAUti v2.6.7 input consisted of the alignment of all SNPs in the non-repetitive, prophage-free, non-recombinant core genome. Tip-dates were defined as the year of isolation of the strains in our panel. We selected a generalized time-reversible (GTR) substitution model with an estimated proportion of invariable sites and four different categories of rate heterogeneities. We furthermore selected an uncorrelated exponentially distributed relaxed molecular clock [35] and a coalescent Bayesian Skyline plot tree prior [36]. The demographic and clock models we selected here are based on previous beast2 analyses into *Salmonella* Paratyphi A [37, 38], which performed in-depth model selection using both path sampling and stepping stone sampling approaches to compare the performance of various competing models of evolution. BEAUti xmls were supplemented with the number of invariant sites in the genome by manually specifying the amount of invariant A, C, T and G nucleotides. Analysis was performed in beast2 using five independent chains of 500 million Markov chain Monte Carlo (MCMC) generations, with samples taken every 50 000 MCMC generations. Refer to File S1 for the xml file used in the beast2 analysis. Log files were checked in Tracer v.1.7.2 [39] for proper mixing and convergence, and to see whether the used chain length produced an effective sampling size (ESS) larger than 400 for all parameters (which indicates sufficient sampling). LogCombiner v2.6.7 was then used to combine tree and log files of the five independent beast2 runs, after having removed a 10 % burn-in from each run. TreeAnnotator v2.6.7 was used to summarize the posterior sample of time-trees into a maximum clade credibility tree using median node heights.

We assessed the reliability of our estimates of divergence times and timescales by conducting a date-randomization test [40]. This test consists of repeating the beast2 analysis 20 times with identical model settings with randomly reshuffled sampling dates. Date randomization of our beast2 xml file was done with the R package Tipdatingbeast v1.1-0 [41] in R v4.1.2.

## Results

### 2020 outbreak of fluoroquinolone-resistant paratyphoid fever in Phnom Penh

**Fig. 1.**
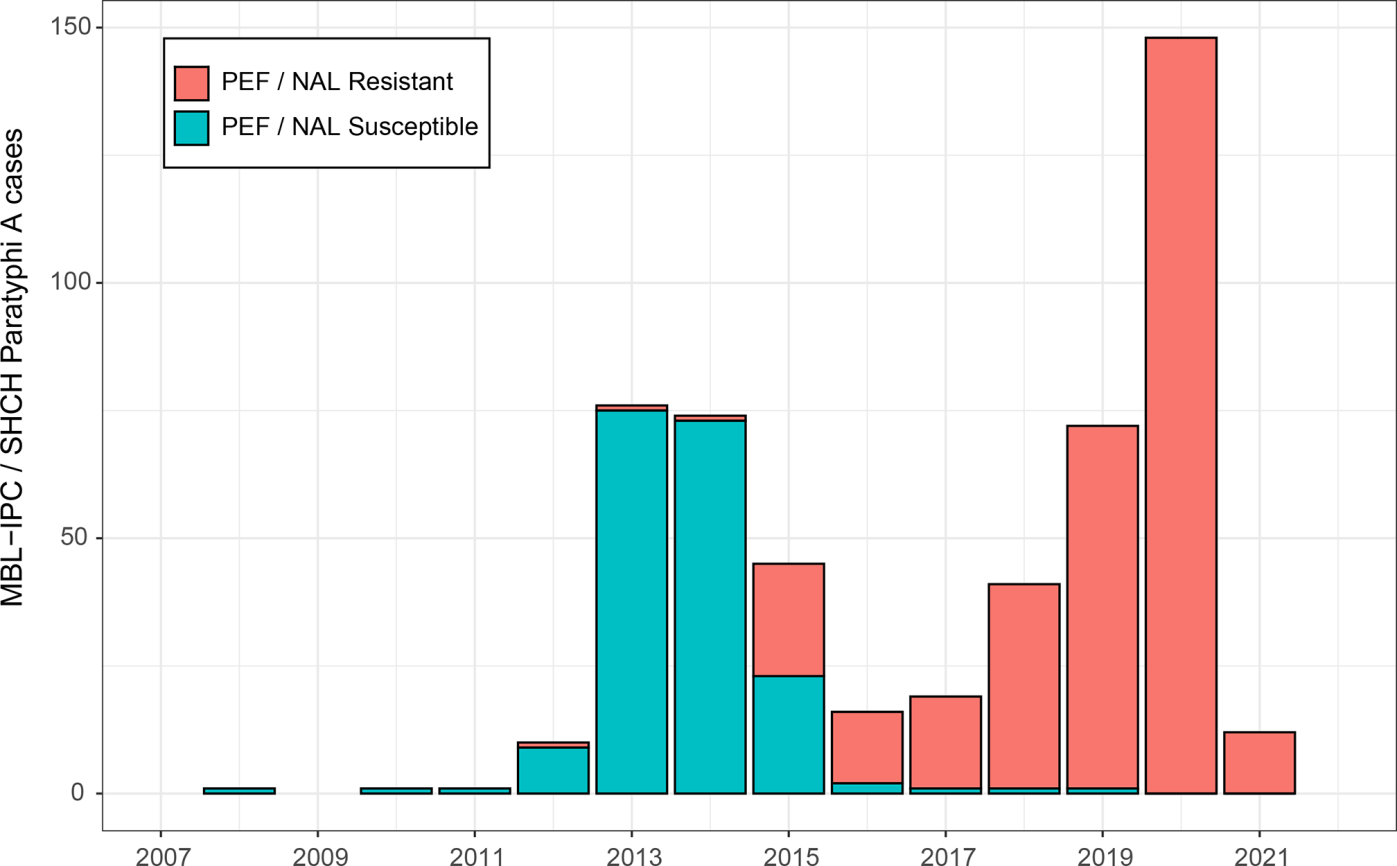
Epidemic curve of all 516 blood-culture-confirmed cases of Paratyphi A identified between 2008 and 2021 at both the MBL-IPC and SHCH. The epidemic curve is annotated with clinical fluoroquinolone-resistance screening results. Clinical fluoroquinolone-resistance screening was performed using nalidixic acid (NAL; 30 µg) disc diffusion until the end of 2009, when the preferred predictor was updated by EUCAST to PEF (5 µg) disc diffusion. The patient catchment area of both the MBL-IPC and SHCH (Phnom Penh and the surrounding provinces) has remained similar throughout the study period.

In early 2020, the MBL-IPC isolated an unusually high number of fluoroquinolone-resistant Paratyphi A strains during its routine diagnostic microbiology activities. A total of 32 Paratyphi A isolates were grown from blood cultures of patients who presented at six different public and private hospitals and clinics. These health centres are located throughout Phnom Penh and rely on the MBL-IPC for medical and biological analyses. The MBL-IPC’s 2020 isolation of fluoroquinolone-resistant Paratyphi A strains was fourfold higher than the mean of the four previous years (2016–2019). Of note, during 2020, not a single fluoroquinolone-susceptible Paratyphi A strain was isolated by the MBL-IPC. After notifying the Cambodian CDC of the rise in fluoroquinolone-resistant Paratyphi A isolation, the MBL-IPC corroborated its findings by reaching out to Sihanouk Hospital Center of HOPE (SHCH), a non-governmental referral hospital for adults in Phnom Penh, which has been performing bloodstream infection surveillance among adult patients in Phnom Penh since 2007. The SHCH observed a similar 2020 peak in the epidemic curve of fluoroquinolone-resistant Paratyphi A (Fig. 1).

### Phenotypic AST and *in silico* detection of resistance mechanisms

All 32 MBL-IPC isolates of the 2020 Cambodian Paratyphi A outbreak were resistant to the fluoroquinolone antibiotics PEF and CIP, and susceptible to all other tested antibiotics including β-lactams, aminoglycosides and macrolides. Phenotypic AST results are summarized in Table S2. This resistance phenotype was largely in agreement with the AST results of 114 additional 2020 SHCH isolates of the same Cambodian Paratyphi A outbreak (Table 1). All 114 SHCH isolates were PEF and CIP resistant, though additional resistance was demonstrated to AZI (*N*=1) and CHL (*N*=13) (Table S2). Consequently, the Cambodian Paratyphi A population remained 100 % susceptible to both the former first-line drugs AMP/TRS and alternative drugs that can be used for treatment of fluoroquinolone-resistant Paratyphi A (CTA, CTR).

**Table 1.** Antibiotic resistance rates of 146 Paratyphi A strains isolated during the 2020 Phnom Penh outbreak at both the MBL-IPC (*N*=32) and SHCH (*N*=114)

Antibiotic class	Antibiotic	2020 Paratyphi A (*N*=146) resistance rate (%)	MIC range (mg l^−1^)
Penicillins	Ampicillin (AMP)	0.0	≤4
3GC	Cefixime (CIX)	0.0	≤0.5
	Ceftazidime (CTZ)	0.0	≤0.5
	Cefotaxime (CTA)	0.0	≤0.5
	Ceftriaxone (CTR)	0.0	≤0.5
4GC	Cefepime (CEP)	0.0	≤1
Carbapenems	Meropenem (MEM)	0.0	≤0.06
Aminoglycosides	Gentamicin (GEN)	0.0	≤2
Macrolides	Azithromycin (AZI)	0.7	8–32
2GFQ	Ciprofloxacin (CIP)	100	0.5–4
	Pefloxacin (PEF)	100	nd*
4GFQ	Gatifloxacin (GAT)	nd†	0.5–2
Others	Trimethoprim–sulfamethoxazole (TRS)	0.0	≤1/19
	Chloramphenicol (CHL)	8.9	≤8–16

3GC, Third-generation cephalosporin; 4GC, fourth-generation cephalosporin; 2GFQ, second-generation fluoroquinolone; 4GFQ, fourth-generation fluoroquinolone; MIC, minimum inhibitory concentration; nd, not determined.

*PEF-based fluoroquinolone-resistance screening was performed using disc diffusion.

†GAT EUCAST breakpoints for *Salmonella* spp. are currently not available.

The resistance genotype predictions made by AMRFinderPlus for the 32 Paratyphi A strains isolated and sequenced by the MBL-IPC were in agreement with all defined resistance phenotypes of these strains. Fluoroquinolone drugs inhibit the action of bacterial DNA gyrase and topoisomerase IV, two essential chromosome-encoded enzymes required for bacterial DNA replication. The four genes encoding the subunits of these tetrameric enzymes (*gyrA* and *gyrB* for DNA gyrase and *parC* and *parE* for topoisomerase IV) can acquire resistance mutations in specific ‘quinolone resistance determining regions’ (QRDR) [42]. Here, *in silico* detection of resistance mechanisms revealed all 32 MBL-IPC Paratyphi A outbreak strains carried the chromosomal *gyrA*-S83F mutation, which causes a serine to phenylalanine amino acid substitution at codon 83 (Table S2). AMRFinderPlus did not detect any other AMR genes in the 32 sequenced Paratyphi A strains. AMRFinderPlus results were very consistent, with 100 % (32/32) of phenotypic susceptibility tests predicted by the resistance genotype.

### Comparative phylogenomic investigation

**Fig. 2.**
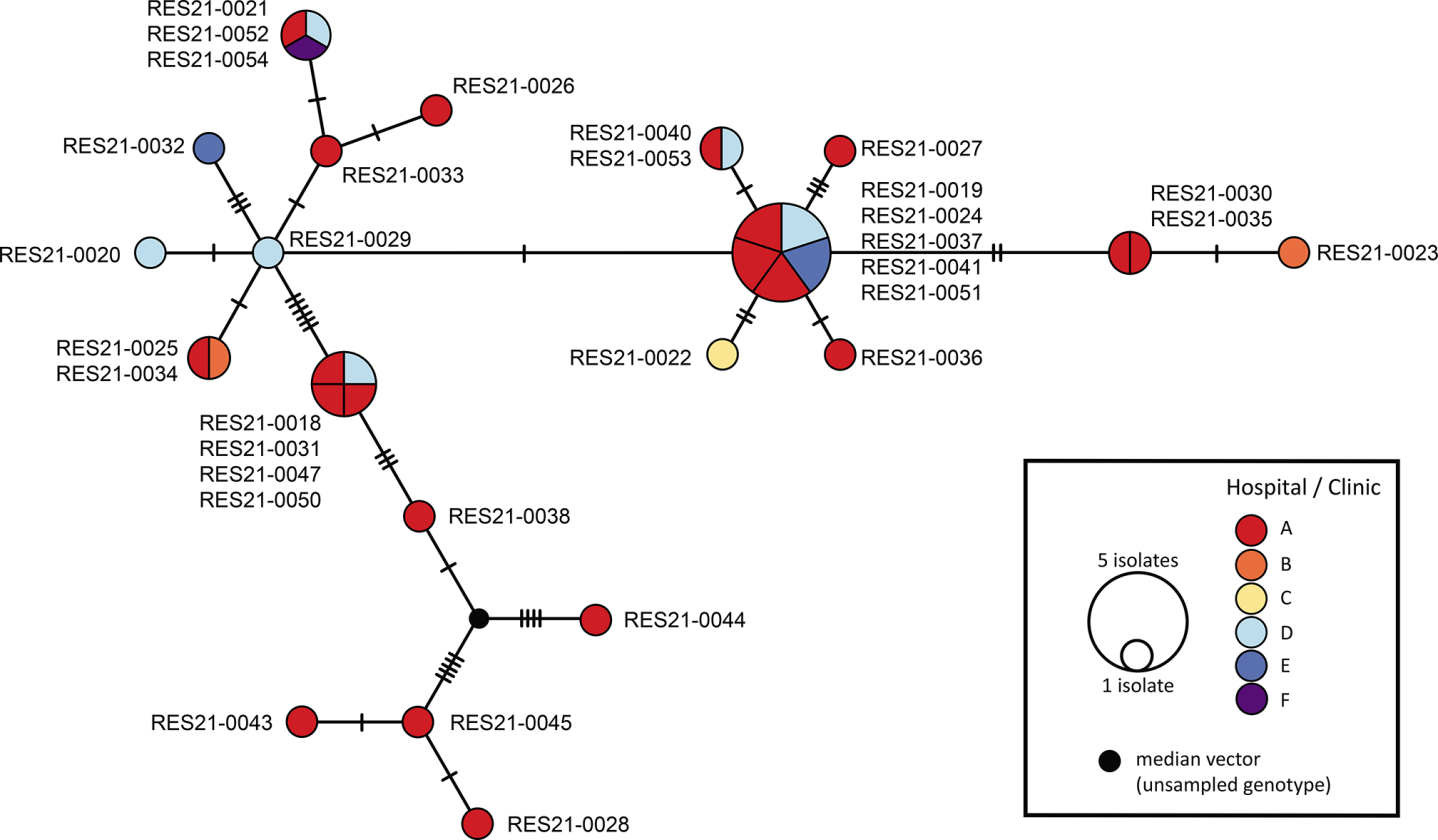
Phylogenetic network showing patterns of descent among the 32 Paratyphi A strains isolated by the MBL-IPC in relation to the Phnom Penh hospital or clinic where patients presented. The median-joining network is based on 40 SNP differences detected across the whole core genome. Each circle represents a unique haplotype, and the size of the circle is proportional to the number of isolates sharing that type. Haplotypes represent variations of a genome in a bacterial population that evolves through the gradual accumulation of mutations. Hatch marks on the branches represent the number of mutational steps between haplotypes. Colour codes represent different hospitals and clinics, as shown in the key.

Genome sequence reads of the 32 Cambodian Paratyphi A 2020 outbreak strains were aligned to the Paratyphi A strain ATCC 9150 reference chromosome and, after excluding all variation detected in repetitive, recombinant and prophage regions, we detected a total diversity of just 40 chromosomal SNPs uniformly distributed along the 4.5 Mb bacterial chromosome. A total of 20 clones (unique genomes) were discerned among the 32 isolates (Fig. 2). Even though Paratyphi serovars are known to be genetically monomorphic bacterial populations [37], the low mean pairwise SNP distance between the outbreak isolates suggested they belonged to an epidemic clonal expansion of an earlier, introduced strain.

The Paratype SNP-based genotyping scheme assigned the 32 sequenced MBL-IPC isolates to Paratyphi A sub-lineage 2.3.1 (formerly known as lineage C5) [21]. To provide appropriate genetic context for interpreting the origin of the studied outbreak of enteric fever, we included in our analysis a selection of Illumina sequence reads of other Paratyphi A lineage 2.3 strains. To achieve this, we filtered down all publicly available lineage 2.3 Paratyphi A read-sets (*n*=526) to a manageable number (*n*=256) that still reflects the breadth of the serovar’s lineage 2.3 diversity. The 270 read-sets excluded during this Treemmer pruning step are detailed in Table S3. The final panel (*n*=256) included Paratyphi A isolates from ten published studies, spanning 22 countries and covering a timeline between 1949 and 2020 (Table S1). Of note, the final panel included all (*n*=76) previously sequenced [6, 37, 38, 43] Cambodian lineage 2.3 Paratyphi A strains.

We did not detect any recombined segments in any isolate of this filtered-down lineage 2.3 panel, supporting a highly clonal population structure for the lineage that has predominantly evolved through the accumulation of vertically inherited mutations, which also aligns with previous findings [37]. We detected 920 SNP differences across the whole core genome of lineage 2.3 and used this diversity to both infer a time-tree and estimate divergence times for key clades. Our Bayesian phylogenetic analysis estimated a mean genome wide substitution rate of 1.41×10^−7^ per site per year [95 % highest posterior density (HPD) interval 1.12×10^−7^ to 1.71×10^−7^], corresponding to the accumulation of 0.65 SNPs per bacterial chromosome per year (95 % HPD interval 0.51 to 0.78), aligning with previous findings [37, 44]. To test the validity of this discovered temporal signal in the data, we performed a date-randomization test, which indicated our data had substantial temporal structure (Fig. S1).

**Fig. 3.**
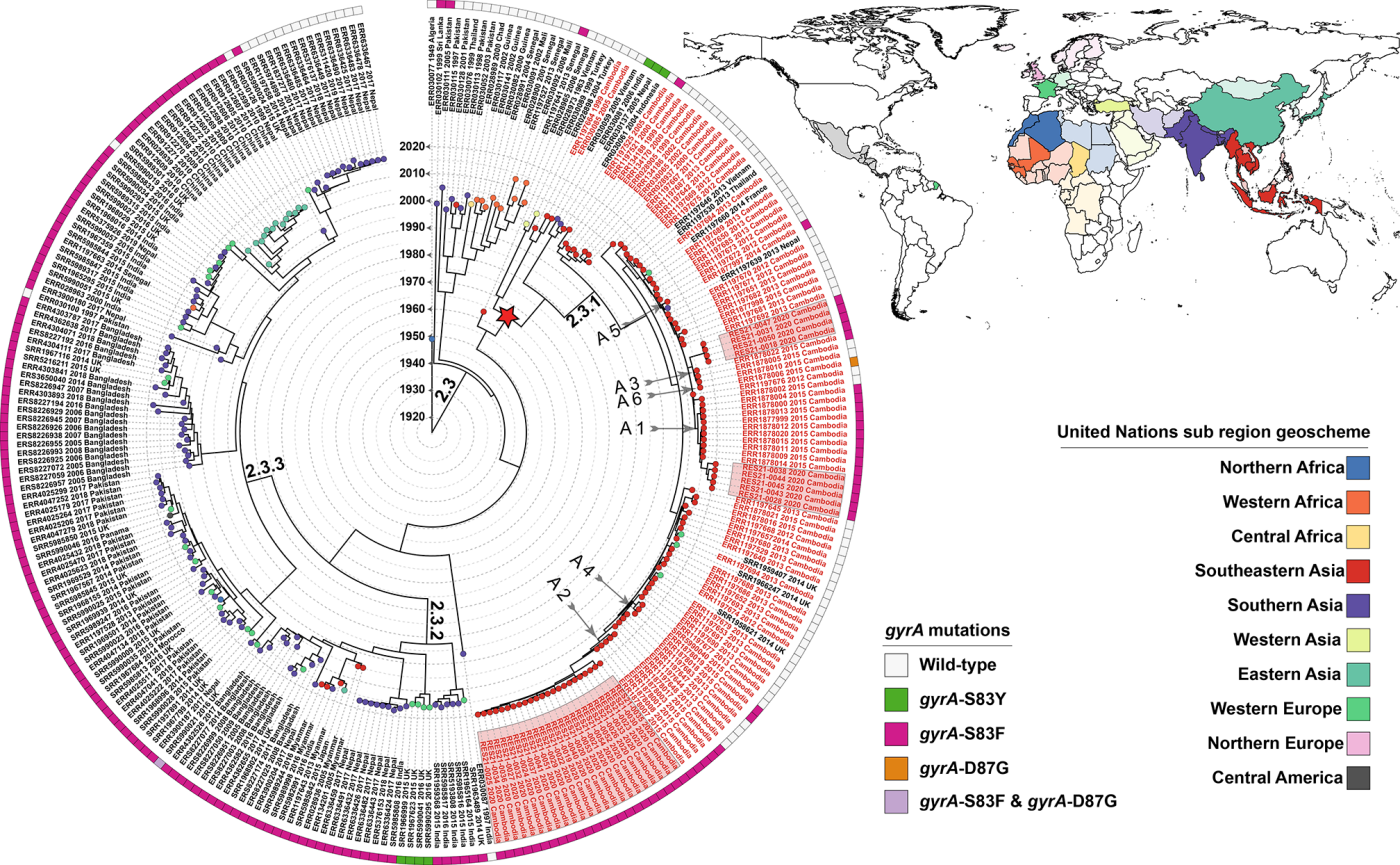
Time-tree of 288 Paratyphi A lineage 2.3 isolates detailing relatedness, geographical origin, chromosomal *gyrA* resistance mutations and the classification of all analysed strains. The maximum clade credibility beast2 tree is based on 920 SNP differences detected across the whole core genome of the isolate panel and was visualized using iTOL v6.5.3 [57]. Paratype [21] (sub-)lineages are annotated on the tree branches. Tip labels of Cambodian isolates are coloured red. The labels highlighted with a pink background represent the studied 2020 MBL-IPC Phnom Penh outbreak isolates. The timing of the transmission event that introduced Paratyphi A lineage 2.3 into Cambodia is annotated as a red star in the phylogeny. The leaves on the tree are coloured according to the geographical origin of the isolates: we classified countries into United Nations sub-regions to limit the number of colours in the phylogeny. The different sub-regions are illustrated in the map using light colours, while countries included in the studied panel belonging to that sub-region are displayed in dark colours. Vector map data of administrative boundaries of countries was obtained from Natural Earth and visualized using qgis v.2.18.13 [58]. The *gyrA* resistance conferring mutations detected in sequence isolates are visualized in the ring around the time-tree. Six discussed independent *gyrA*-S83F acquisition events are annotated on the tree using arrows.

Reconstruction of the lineage 2.3 Paratyphi A time-tree over a period of 105 years since its most recent common ancestor (MRCA) allowed us to date the historical global transmission event that introduced the pathogen in Cambodia to 1965 (95 % HPD interval: 1952–1983) (Fig. 3). We furthermore observed Cambodian isolates are the most closely related to Vietnamese isolate ERR028973, with which they share a common ancestor in 1957 (95 % HPD interval: 1944–1962). As our time-tree did not encompass 270 additional publicly available lineage 2.3 Paratyphi A read-sets (Treemmer pruning), we inferred an additional ML tree with all read-sets available in sequence repositories (*n*=526) to confirm this association with historic Vietnamese strains isolated in 1963 (Fig. S2).

The time-tree furthermore indicated that since the introduction event, sub-lineage 2.3.1 has become the dominant sub-lineage in Cambodia [*t*_MRCA_2.3.1=1993 (95 % HPD interval: 1985–1998)]. Inspection of the time-tree revealed the 2020 Cambodian outbreak strains are the descendants of a previously described 2013–2015 outbreak of 2.3.1 Paratyphi A infections reported in Cambodian patients and American, European and Japanese travellers returning from Cambodia [38].

It is clear that, since its first detection in 1999, sub-lineage 2.3.1 has remained largely susceptible to fluoroquinolone antibiotics until 2015 (Fig. 3). Close inspection of the time-tree revealed sub-lineage 2.3.1 acquired chromosomal resistance to fluoroquinolone antibiotics during six separate events through the independent acquisition of *gyrA*-S83F*/gyrA*-D87G mutations (Table 2, Fig. 3). The 2020 fluoroquinolone-resistant Cambodian Paratyphi A outbreak isolates studied here are the descendants of three of these ancestor strains that expanded clonally after the acquisition of resistance-conferring mutations (acquisition events A1, A2 and A3).

**Table 2.** Overview of all Paratyphi A sub-lineage 2.3.1 *gyrA*-S83F*/gyrA*-D87G acquisition events Isolates sequenced in this study are highlighted in bold. The timing of acquisition events was inferred by estimating the *t*_MRCA_ of all sequenced isolates that descended from an ancestor strain that acquired the resistance-conferring mutation. The uncertainty around the timing is reflected in the 95 % HPD interval. As isolates ERR1877997 and ERR1878006 represent singletons, the timing of acquisition event 5/6 is reflected by the isolation year of these isolates (2014 and 2015, respectively). Note the ERR1878006 *gyrA* mutation was incorrectly called as *gyrA*-S83F in another publication [6].

Acquisition event	*gyrA* mutation	Descendant strain count	*t*_MRCA_	95 % HPD interval	Isolate
A 1	*gyrA*-S83F	15	Oct 2012	Mar 2011–Jan 2014	ERR1878004, ERR1878000, ERR1878013, ERR1877999, ERR1878012, ERR1878020, ERR1878015, ERR1878011, ERR1878009, ERR1878014, **RES21-0038**, **RES21-0044**, **RES21-0045**, **RES21-0043**,**RES21-0028**
A 2	*gyrA*-S83F	28	Jul 2013	Nov 2011–Jul 2014	ERR1878017, ERR1878003, ERR1878001, ERR1878008, ERR1878007, **RES21-0035, RES21-0030**, **RES21-0023**, **RES21-0033**, **RES21-0026**, **RES21-0052**, **RES21-0054**,**RES21-0021**, **RES21-0053**, **RES21-0020**, **RES21-0040**, **RES21-0019**, **RES21-0022**, **RES21-0029**, **RES21-0051**, **RES21-0041**, **RES21-0037**, **RES21-0024**, **RES21-0032**, **RES21-0027**, **RES21-0036**, **RES21-0034**, **RES21-0025**
A 3	*gyrA*-S83F	5	Sep 2013	Aug 2011–Jun 2014	**RES21-0047**, **RES21-0031**, **RES21-0050**, **RES21-0018**, ERR1878022
A 4	*gyrA*-S83F	2	Apr 2014	Jul 2011–Jun 2014	ERR1878018, ERR1878019
A 5	*gyrA*-S83F	1	2014	na	ERR1877997
A 6	*gyrA*-D87G	1	2015	na	ERR1878006

NA, not applicable.

**Fig. 4.**
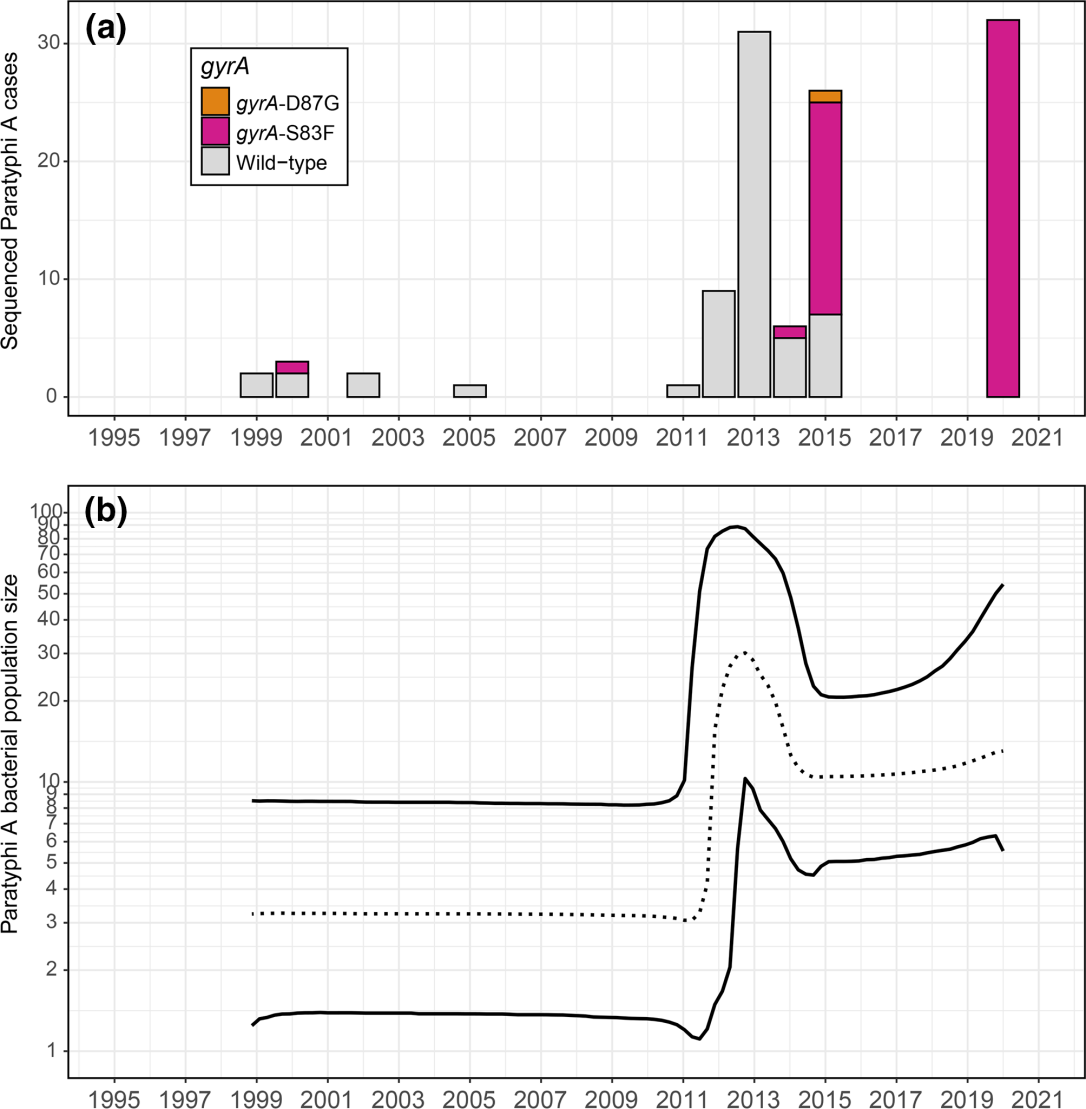
The demographic history of Cambodian Paratyphi A sub-lineage 2.3.1. (a) All 113 sequenced 2.3.1 isolates annotated with their *gyrA* resistance conferring mutations. (b) Bayesian Skyline plot that shows changes in the bacterial effective population size (*N*_*e*_) through time since 1999, based on 95 SNP differences detected across the whole core genome of the sequenced 2.3.1 isolate panel (*n*=113). This plot allows us to discuss relative increases or decreases to the bacterial population size through time. The central dashed line represents the median bacterial population size with its 95 % central posterior density (CPD) interval represented by the upper and lower lines. Note the *y*-axis is on a log scale.

Reconstruction of the demographic history of all sequenced Paratyphi A sub-lineage 2.3.1 isolates (Fig. 4a) revealed a sharp increase in the bacterial population size in 2012–2013, followed by a subtle decrease in 2014–2015 (Fig. 4b). This peak in the inferred bacterial population size aligned with the 2013–2015 community outbreak during which fluoroquinolone resistance emerged within the susceptible wild-type population and spread rapidly. Consequently, the frequency of fluoroquinolone-resistant blood-culture-confirmed cases increased dramatically in the subsequent years (2016–2020) (Fig. 1).

### Screening food isolates

We attempted to identify geographical point sources linked to the 2020 outbreak of paratyphoid fever by screening a collection of 83 *Salmonella* spp. strains isolated from Phnom Penh food samples between 2018 and 2020. This *Salmonella* spp. panel was collected during the LEFS-IPC’s routine microbiological control of food samples that were submitted between 2020 and the 2 years preceding the outbreak. However, Paratyphi A-specific gene combination qPCR assays did not identify any Paratyphi A isolates in this *Salmonella* spp. collection. Only non-typhoidal salmonellae were identified (data not shown).

## Discussion

The isolation of an unusually high frequency of fluoroquinolone-resistant Paratyphi A strains from Phnom Penh in early 2020 drove our laboratory to support a public-health investigation. We analysed the extant genetic diversity of the 32 MBL-IPC 2020 outbreak strains by sequencing and comparing their genomes to 256 closely related lineage 2.3 isolates and inferring a phylogenetic time-tree that describes the evolution of the Paratyphi A population from a single common ancestor in the past. Our comparative genomic analysis revealed the 2020 outbreak strains descended from a previously described 2013–2015 outbreak of lineage 2.3.1 Paratyphi A infections [6, 38, 45, 46]. While the original genomic analysis of the 2013–2015 outbreak was limited to isolates from 2013 to early 2014 that were largely pan-susceptible to antibiotics [38], subsequent microbiological analysis of strains isolated later in the outbreak (2014–2015) indicated resistance to fluoroquinolone drugs was emerging [6].

We observed a definite shift in the antimicrobial susceptibility of paratyphoid salmonellae. Our analysis confirmed sub-lineage 2.3.1 remained largely susceptible to fluoroquinolone drugs until 2015, and revealed sub-lineage 2.3.1 acquired chromosomal resistance to these drugs during six separate events through the independent acquisition of *gyrA*-S83F and *gyrA*-D87G mutations between late 2012 and 2015. This observation is in sharp contrast to widespread resistance to these drugs found in South Asian sub-lineages 2.3.2 and 2.3.3, which emerged noticeably earlier (Fig. 3). The rapid acquisition rate of resistance-conferring mutations in the Cambodian Paratyphi A population over a 3 year period is suggestive of a strong selective pressure on that population [47]. As Paratyphi A is restricted to the human host, it is clear that this selective pressure is linked with fluoroquinolone use during the 2013–2015 ‘community outbreak’ [45, 46] when the bacterial effective population size peaked (Fig. 4b). The emergence of fluoroquinolone resistance during this peak was rapidly followed by the replacement of the original susceptible wild-type Paratyphi A population, which led to a dramatic increase of fluoroquinolone-resistant blood-culture-confirmed cases in the subsequent years (2016–2021), to the current proportion of 100 % fluoroquinolone resistance (Fig. 1).

The appearance of widespread chromosomal resistance to fluoroquinolones since 2016 has reduced currently recommended therapeutic options of Cambodian patients to macrolides (like AZI) or third-generation cephalosporins (like CTA and CTR) [9]. While there are reports of emerging resistance against these alternative treatment options in paratyphoid salmonellae [48, 49], our AST results and those of other studies [6] reveal the Paratyphi A population in Cambodia remains largely unaffected by these worrying developments. The 2020 outbreak isolates were found to be 100 % susceptible to all tested cephalosporins (CIX, CTZ, CTA, CTR and CEP) and the resistance rate to AZI was low (0.7 %). Phenotypic AST results furthermore revealed the Cambodian Paratyphi A population remained 100 % susceptible to the former first-line drugs AMP and TRS, which suggests the role of using these conventional anti-typhoid drugs in the treatment of Cambodian Paratyphi A infections could be re-evaluated [50]. In contrast, the third, former first-line drug, CHL, is known to expose patients to serious toxicity to the bone marrow [1], which has made it virtually obsolete in many western countries. As our analysis revealed the Cambodian 2020 outbreak isolates to be 8.9 % resistant to the drug, CHL should be set aside, especially as the other former first-line drugs AMP and TRS have lower toxicity and retained their effectiveness against Paratyphi A in Cambodia.

Antibiotic misuse and overuse is common practice in the region, where there is a high burden of infectious diseases, access to antibiotics is largely unrestricted, and self-medication with drug-cocktails including broad-spectrum antibiotics is common [51]. Furthermore, national treatment guidelines for antimicrobial use in infectious diseases based on the local ecology of bacterial resistance are lacking. Our findings point towards the negative consequences of misuse of fluoroquinolones, the most effective drugs available for the management of enteric fever. It is imperative that antibiotics are used judiciously to prevent further escalation of the global crisis of antibiotic resistance. Improved control of antibiotic access requires both bolstered enforcement of existing pharmaceutical legislation and enacting new regulatory frameworks that put a halt to over-the-counter non-prescription dispensing of antibiotic agents. Additionally, as pervasive antibiotic misuse is in part driven by misconceptions about antibiotics, educational programmes should be implemented to change local health-seeking behaviours.

Interestingly, inspection of our time-tree revealed Paratyphi A was introduced into Cambodia in 1965 [95 % HPD: 1952–1983], during a dark chapter of Cambodian history. After having gained its independence in 1953, Cambodia struggled to remain neutral in a cold war-era world marked by proxy wars in South-East Asia. In 1964, the Vietnam War spilled over into neighbouring Cambodia, which exacerbated into the Cambodian Civil War (1967) that ended with the establishment of the Khmer Rouge regime (1975). During the Khmer Rouge’s genocidal reign, the entire population of Phnom Penh was forcibly displaced into labour camps, which led to the deaths of up to two million people. In 1979, Cambodia was liberated from Khmer Rouge by Vietnam, which occupied the country until 1989 [52]. We posit here this succession of conflicts created favourable conditions for outbreaks of various infectious diseases transmitted through the faecal–oral route. War significantly increases the risk of paratyphoid fever through degraded living conditions, the breakdown of hygiene and sanitary infrastructure, and poor access to safe food and water [53]. To investigate the possible origin of Cambodian Paratyphi A, we furthermore compared all 108 sequenced Cambodian isolates to the genomes of 21 other countries. This revealed Cambodian Paratyphi A isolates were the most closely related to a Vietnamese strain (ERR028973) isolated in 1963, with which they share a common ancestor in 1957 [95 % HPD interval: 1944–1962]. It is tempting to suggest Paratyphi A might have been introduced from Vietnam given known historic population movements linked to the second Indochina war and the subsequent 10 year long occupation by Vietnam. However, the limited amount of available historic isolates sampled from South-East Asia limits our ability to support this claim with any certainty.

WGS can delineate community outbreaks of foodborne bacteria by characterizing transmission events and confirming contamination sources [54]. As all 2020 Paratyphi A isolates analysed here were de-identified to preserve patient privacy, we could not carry out proper exposure investigations with standardized questionnaires and/or patient interviews to identify potential common sources of exposure. We nevertheless attempted to identify point sources by screening a collection of *Salmonella* spp. strains isolated from Phnom Penh food samples collected in the years preceding the 2020 outbreak. While our screening effort only identified non-typhoidal salmonellae, the screened collection only encompassed isolates obtained from food samples voluntarily submitted by the LEFS-IPC clients. These clients are predominantly large food businesses, and don’t include street-food vendors and informal markets where food mishandling practices are prevalent and food-safety regulations are less well followed [55].

The rapid emergence and spread of fluoroquinolone resistance in the Paratyphi A population in Cambodia described here highlights the importance of regional AMR genomic surveillance in low- and middle-income countries (LMIC). The barriers to introducing WGS and to translating genomic AMR surveillance into public-health action in LMIC include high initial investment, the cost of laboratory and computing infrastructure, expertise, and training requirements. However, the cost-effectiveness of WGS is changing rapidly, providing an opportunity to narrow the gaps in knowledge and technology in LMIC and strengthen basic capability to use WGS for AMR surveillance to enhance public health [56].

## Supplementary Data

Supplementary material 1

Supplementary material 2

## References

[1] Crump JA, Sjölund-Karlsson M, Gordon MA, Parry CM (2015). Epidemiology, clinical presentation, laboratory diagnosis, antimicrobial resistance, and antimicrobial management of invasive *Salmonella* infections. Clin Microbiol Rev.

[2] Stanaway JD, Reiner RC, Blacker BF, Goldberg EM, Khalil IA (2019). The global burden of typhoid and paratyphoid fevers: a systematic analysis for the Global Burden of Disease Study 2017. Lancet Infect Dis.

[3] Public Health Laboratory Service (1978). Waterborne infectious disease in Britain. J Hyg.

[4] Ochiai RL, Wang X, von Seidlein L, Yang J, Bhutta ZA (2005). *Salmonella* Paratyphi A rates, Asia. Emerg Infect Dis.

[5] Sood S, Kapil A, Dash N, Das BK, Goel V (1999). Paratyphoid fever in India: an emerging problem. Emerg Infect Dis.

[6] Kuijpers LMF, Phe T, Veng CH, Lim K, Ieng S (2017). The clinical and microbiological characteristics of enteric fever in Cambodia, 2008–2015. PLoS Negl Trop Dis.

[7] Simanjuntak CH, Totosudirjo H, Haryanto P, Suprijanto E, Paleologo FP (1991). Oral immunisation against typhoid fever in Indonesia with Ty21a vaccine. Lancet.

[8] Holt KE, Thomson NR, Wain J, Phan MD, Nair S (2007). Multidrug-resistant *Salmonella enterica* serovar Paratyphi A harbors IncHI1 plasmids similar to those found in serovar Typhi. J Bacteriol.

[9] WHO (2003). Background Document: the Diagnosis, Treatment and Prevention of Typhoid Fever.

[10] Mirza SH, Beeching NJ, Hart CA (1995). The prevalence and clinical features of multi-drug resistant *Salmonella typhi* infections in Baluchistan, Pakistan. Ann Trop Med Parasitol.

[11] Mandal S, Mandal MD, Pal NK (2006). Antibiotic resistance of *Salmonella enterica* serovar Paratyphi A in India: emerging and reemerging problem. J Postgrad Med.

[12] Butt T, Ahmad RN, Salman M, Kazmi SY (2005). Changing trends in drug resistance among typhoid salmonellae in Rawalpindi, Pakistan. East Mediterr Health J.

[13] Chandel DS, Chaudhry R, Dhawan B, Pandey A, Dey AB (2000). Drug-resistant *Salmonella enterica* serotype Paratyphi A in India. Emerg Infect Dis.

[14] Woods CW, Murdoch DR, Zimmerman MD, Glover WA, Basnyat B (2006). Emergence of *Salmonella enterica* serotype Paratyphi A as a major cause of enteric fever in Kathmandu, Nepal. Trans R Soc Trop Med Hyg.

[15] Maskey AP, Day JN, Phung QT, Thwaites GE, Campbell JI (2006). *Salmonella enterica* serovar Paratyphi A and *S. enterica* serovar Typhi cause indistinguishable clinical syndromes in Kathmandu, Nepal. Clin Infect Dis.

[16] Grimont P, Weill F-X (2007). Antigenic Formulae of the Salmonella Serovars, 9th edn.

[17] Nair S, Patel V, Hickey T, Maguire C, Greig DR (2019). Real-time PCR assay for differentiation of typhoidal and nontyphoidal *Salmonella*. J Clin Microbiol.

[18] EUCAST (2021). Breakpoint Tables for Interpretation of MICs and Zone Diameters, version 11.0.

[19] Bolger AM, Lohse M, Usadel B (2014). Trimmomatic: a flexible trimmer for Illumina sequence data. Bioinformatics.

[20] Menardo F, Loiseau C, Brites D, Coscolla M, Gygli SM (2018). Treemmer: a tool to reduce large phylogenetic datasets with minimal loss of diversity. BMC Bioinformatics.

[21] Tanmoy AM, Hooda Y, Sajib MSI, da Silva KE, Iqbal J (2022). Paratype: a genotyping tool for *Salmonella* Paratyphi A reveals its global genomic diversity. Nat Commun.

[22] Seemann T (2022). Shovill v1.1.0. https://github.com/tseemann/shovill.

[23] Feldgarden M, Brover V, Gonzalez-Escalona N, Frye JG, Haendiges J (2021). AMRFinderPlus and the reference gene catalog facilitate examination of the genomic links among antimicrobial resistance, stress response, and virulence. Sci Rep.

[24] Seemann T (2022). Snippy v4.6.0. https://github.com/tseemann/snippy.

[25] Li H, Durbin R (2010). Fast and accurate long-read alignment with Burrows-Wheeler transform. Bioinformatics.

[26] Danecek P, Bonfield JK, Liddle J, Marshall J, Ohan V (2021). Twelve years of SAMtools and BCFtools. Gigascience.

[27] Garrison E, Marth G (2012). Haplotype-based variant detection from short-read sequencing. *arXiv*:12073907 [q-bio]. 2012 [cited 18 Feb 2022]. http://arxiv.org/abs/1207.3907.

[28] Croucher NJ, Page AJ, Connor TR, Delaney AJ, Keane JA (2015). Rapid phylogenetic analysis of large samples of recombinant bacterial whole genome sequences using Gubbins. Nucleic Acids Res.

[29] Arndt D, Grant JR, Marcu A, Sajed T, Pon A (2016). PHASTER: a better, faster version of the PHAST phage search tool. Nucleic Acids Res.

[30] Leigh JW, Bryant D (2015). Popart: full-feature software for haplotype network construction. Methods Ecol Evol.

[31] Bandelt HJ, Forster P, Röhl A (1999). Median-joining networks for inferring intraspecific phylogenies. Mol Biol Evol.

[32] Nguyen L-T, Schmidt HA, von Haeseler A, Minh BQ (2015). IQ-TREE: a fast and effective stochastic algorithm for estimating maximum-likelihood phylogenies. Mol Biol Evol.

[33] Kalyaanamoorthy S, Minh BQ, Wong TKF, von Haeseler A, Jermiin LS (2017). ModelFinder: fast model selection for accurate phylogenetic estimates. Nat Methods.

[34] Bouckaert R, Vaughan TG, Barido-Sottani J, Duchêne S, Fourment M (2019). BEAST 2.5: an advanced software platform for Bayesian evolutionary analysis. PLoS Comput Biol.

[35] Drummond AJ, Ho SYW, Phillips MJ, Rambaut A (2006). Relaxed phylogenetics and dating with confidence. PLoS Biol.

[36] Drummond AJ, Rambaut A, Shapiro B, Pybus OG (2005). Bayesian coalescent inference of past population dynamics from molecular sequences. Mol Biol Evol.

[37] Zhou Z, McCann A, Weill F-X, Blin C, Nair S (2014). Transient Darwinian selection in *Salmonella enterica* serovar Paratyphi A during 450 years of global spread of enteric fever. Proc Natl Acad Sci USA.

[38] Kuijpers LMF, Le Hello S, Fawal N, Fabre L, Tourdjman M (2016). Genomic analysis of *Salmonella enterica* serotype Paratyphi A during an outbreak in Cambodia, 2013-2015. Microb Genom.

[39] Rambaut A, Drummond AJ, Xie D, Baele G, Suchard MA (2018). Posterior summarization in Bayesian phylogenetics using tracer 1.7. Syst Biol.

[40] Duchene S, Duchene DA, Geoghegan JL, Dyson ZA, Hawkey J (2018). Inferring demographic parameters in bacterial genomic data using Bayesian and hybrid phylogenetic methods. BMC Evol Biol.

[41] Rieux A, Khatchikian CE (2017). TIPDATINGBEAST: an R package to assist the implementation of phylogenetic tip-dating tests using BEAST. Mol Ecol Resour.

[42] Jacoby GA (2005). Mechanisms of resistance to quinolones. Clin Infect Dis.

[43] Nair S, Day M, Godbole G, Saluja T, Langridge GC (2020). Genomic surveillance detects *Salmonella enterica* serovar Paratyphi A harbouring *bla*CTX-M-15 from a traveller returning from Bangladesh. PLoS One.

[44] Duchêne S, Holt KE, Weill F-X, Le Hello S, Hawkey J (2016). Genome-scale rates of evolutionary change in bacteria. Microb Genom.

[45] Kuijpers LMF, Veng CH, Sar D, Chung P, Phe T (2015). Ongoing outbreak of *Salmonella enterica* serovar Paratyphi A infections, Phnom Penh, Cambodia. J Infect Dev Ctries.

[46] Vlieghe E, Phe T, De Smet B, Veng CH, Kham C (2013). Increase in *Salmonella enterica* serovar Paratyphi A infections in Phnom Penh, Cambodia, January 2011 to August 2013. Euro Surveill.

[47] Cantón R, Morosini M-I (2011). Emergence and spread of antibiotic resistance following exposure to antibiotics. FEMS Microbiol Rev.

[48] Hassing R-J, Goessens WHF, van Pelt W, Mevius DJ, Stricker BH (2014). *Salmonella* subtypes with increased MICs for azithromycin in travelers returned to The Netherlands. Emerg Infect Dis.

[49] Pokharel BM, Koirala J, Dahal RK, Mishra SK, Khadga PK (2006). Multidrug-resistant and extended-spectrum beta-lactamase (ESBL)-producing *Salmonella enterica* (serotypes Typhi and Paratyphi A) from blood isolates in Nepal: surveillance of resistance and a search for newer alternatives. Int J Infect Dis.

[50] Misra R, Prasad KN, Amrin N, Kapoor P, Singh S (2015). Absence of multidrug resistance in *Salmonella enterica* serotypes Typhi and Paratyphi A isolates with intermediate susceptibility to ciprofloxacin. Trans R Soc Trop Med Hyg.

[51] Om C, Daily F, Vlieghe E, McLaughlin JC, McLaws M-L (2017). Pervasive antibiotic misuse in the Cambodian community: antibiotic-seeking behaviour with unrestricted access. Antimicrob Resist Infect Control.

[52] Schanberg SH (1985). The Death and Life of Dith Pran.

[53] Punda-Polić V, Kraljević KS, Bradarić N (2007). War-associated cases of typhoid fever imported to Split-Dalmatia County (Croatia). Mil Med.

[54] Octavia S, Wang Q, Tanaka MM, Kaur S, Sintchenko V (2015). Delineating community outbreaks of *Salmonella enterica* serovar Typhimurium by use of whole-genome sequencing: insights into genomic variability within an outbreak. J Clin Microbiol.

[55] Thompson L, Vipham J, Hok L, Ebner P (2021). Towards improving food safety in Cambodia: current status and emerging opportunities. Glob Food Sec.

[56] WHO (2020). GLASS Whole-Genome Sequencing for Surveillance of Antimicrobial Resistance.

[57] Letunic I, Bork P (2021). Interactive tree of life (iTOL) v5: an online tool for phylogenetic tree display and annotation. Nucleic Acids Res.

[58] QGIS Development Team (2021). QGIS Geographic Information System. https://www.qgis.org.

