# Supplementary material for: Phylogenomic investigation of an outbreak of fluoroquinolone-resistant Salmonella enterica subsp. enterica serovar Paratyphi A in Phnom Penh, Cambodia
Source: Microb Genom. 2023 Mar 24;9(3):mgen000972. doi: 10.1099/mgen.0.000972 (PMC10132074; doi:10.1099/mgen.0.000972)
Supplement: Supplementary material 1 [file mgen-9-972-s001.pdf]

<u>Figure S1</u>: Date-randomization test. We repeated our BEAST2 analysis 20 times with identical model settings but with randomly reshuffled sampling dates. A dataset is considered to have strong temporal structure if the rate estimate obtained using the genuine sampling times is not contained within the range of values from the randomized permutations.

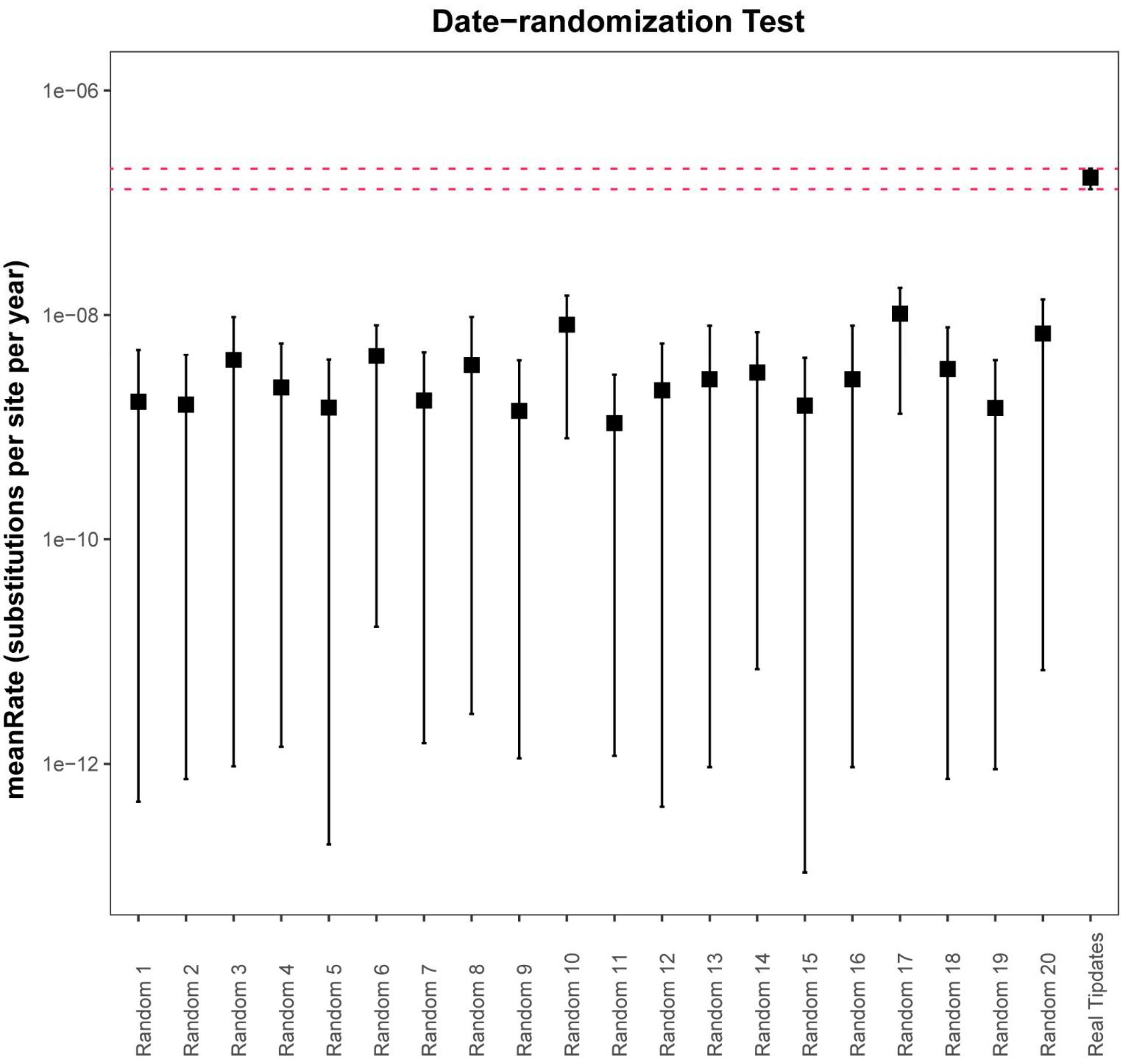

<u>Figure S2</u>: ML-tree of 558 Paratyphi A lineage 2.3 isolates detailing relatedness, geographical origin, and year of isolation of all analyzed strains.

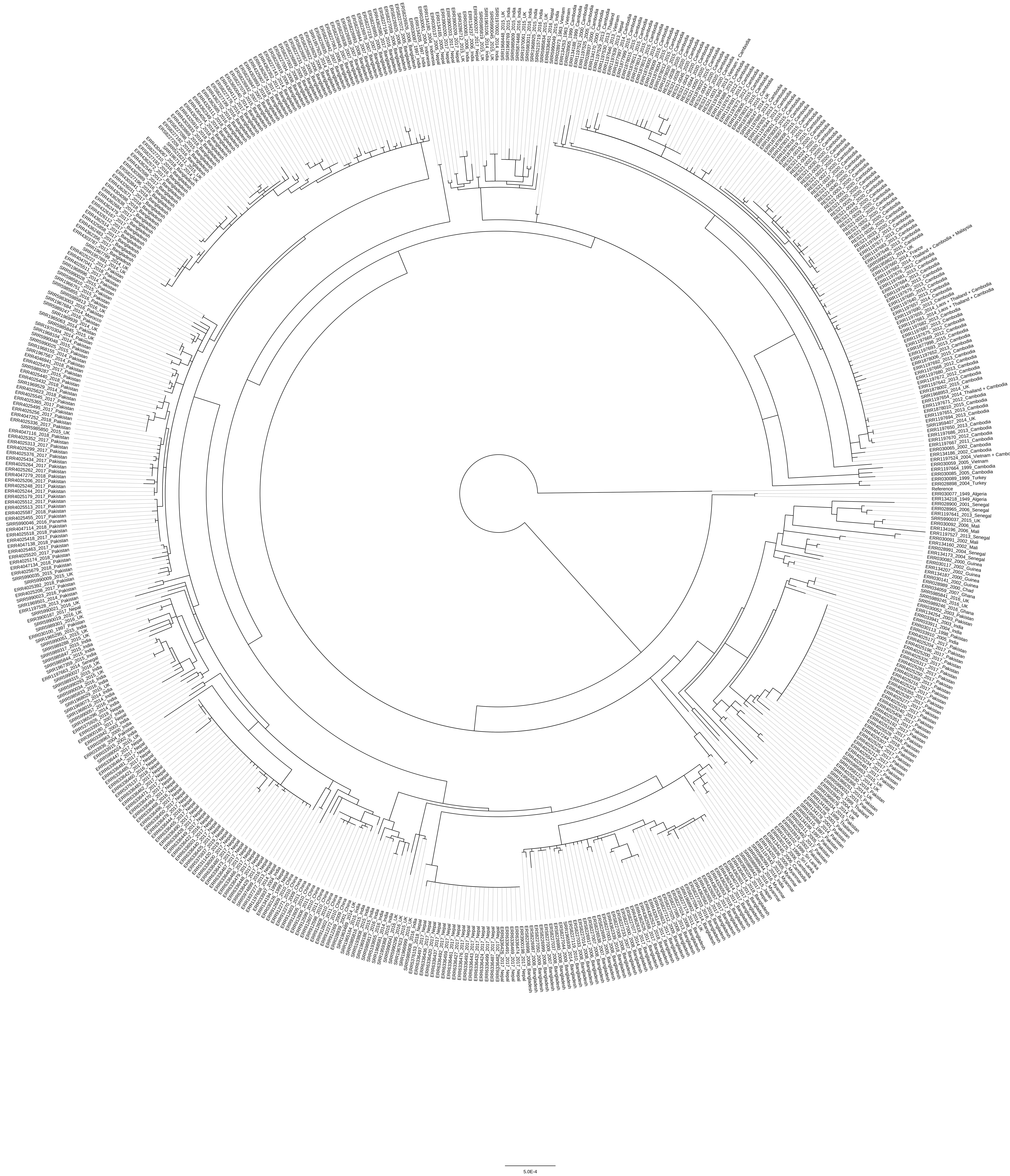
5.0E-4
